# Characteristics of rumen microbiota and *Prevotella* isolates found in high propionate and low methane-producing dairy cows

**DOI:** 10.3389/fmicb.2024.1404991

**Published:** 2024-06-03

**Authors:** Takumi Shinkai, Shuhei Takizawa, Osamu Enishi, Koji Higuchi, Hideyuki Ohmori, Makoto Mitsumori

**Affiliations:** Division of Dairy Cattle Feeding and Breeding Research, Institute of Livestock and Grassland Science, National Agriculture and Food Research Organization, Tsukuba, Ibaraki, Japan

**Keywords:** methane, propionate, dairy cow, rumen microbiota, *Prevotella*

## Abstract

Ruminal methane production is the main sink for metabolic hydrogen generated during rumen fermentation, and is a major contributor to greenhouse gas (GHG) emission. Individual ruminants exhibit varying methane production efficiency; therefore, understanding the microbial characteristics of low-methane-emitting animals could offer opportunities for mitigating enteric methane. Here, we investigated the association between rumen fermentation and rumen microbiota, focusing on methane production, and elucidated the physiological characteristics of bacteria found in low methane-producing cows. Thirteen Holstein cows in the late lactation stage were fed a corn silage-based total mixed ration (TMR), and feed digestion, milk production, rumen fermentation products, methane production, and rumen microbial composition were examined. Cows were classified into two ruminal fermentation groups using Principal component analysis: low and high methane-producing cows (36.9 vs. 43.2 L/DMI digested) with different ruminal short chain fatty acid ratio [(C2+C4)/C3] (3.54 vs. 5.03) and dry matter (DM) digestibility (67.7% vs. 65.3%). However, there were no significant differences in dry matter intake (DMI) and milk production between both groups. Additionally, there were differences in the abundance of OTUs assigned to uncultured *Prevotella* sp., *Succinivibrio*, and other 12 bacterial phylotypes between both groups. Specifically, a previously uncultured novel *Prevotella* sp. with lactate-producing phenotype was detected, with higher abundance in low methane-producing cows. These findings provide evidence that *Prevotella* may be associated with low methane and high propionate production. However, further research is required to improve the understanding of microbial relationships and metabolic processes involved in the mitigation of enteric methane.

## 1 Introduction

Ruminants have a unique digestive system in their foregut that is beneficial for both the animal and rumen microbiota. Feed carbohydrates are digested by rumen microbes to yield short-chain fatty acids (SCFA) in a process known as rumen fermentation, and is associated with the generation of metabolic hydrogen (Guyader et al., 2017). Enteric methane production is the main sink for metabolic hydrogen and is recognized as a considerable contributor to greenhouse gas (GHG) emission (Janssen, 2010; Ungerfeld, 2013; Wattiaux et al., 2019). Therefore, various approaches have been proposed to mitigate methane emission, including direct inhibition of methanogens with chemical inhibitors, feed manipulation to reduce hydrogen production, and maximization of hydrogen consumption (Belanche et al., 2015; Hristov et al., 2015; Martinez-Fernandez et al., 2018; Granja-Salcedo et al., 2019). A comprehensive understanding of ruminal digestion and function will facilitate the development of a holistic and environmentally friendly approach for sustainable ruminant production (Bocquier and González-García, 2010; Wallace et al., 2019).

Rumen microbiota is considered an important factor affecting methane production and feed efficiency (Shabat et al., 2016; Auffret et al., 2018). As the rumen microbiota is different in high- and low-methane-producing cows, microbial composition analysis is necessary to understand the consistent microbial community structure. This microbial information could promote rumen manipulation for environment-friendly methane mitigation (Wallace et al., 2019).

Previous studies using chamber method have shown that the amount of digested dry matter (DM) is a key factor determining the variation in methane production in lactating Holstein cows (Brask et al., 2015). Precise datasets, including those for methane production, feed digestion, and rumen fermentation, are important for elucidating the microbial and functional characteristics of animals (Hill et al., 2016; Hristov et al., 2018). Kittelmann et al. (2014) reported that an abundance of propionate-producing *Quinella* and lactate-producing *Sharpea* in low-methane-producing sheep. Additionally, unidentified *Succinivibrionaceae* and/or *Prevotella* spp. have been detected in low methane-producing cows (Wallace et al., 2015; Danielsson et al., 2017). However, phylogenetic community analysis is sometimes insufficient to understand the functional characteristics of rumen microbiota. For instance, several operational taxonomic units (OTUs) assigned to the genera *Prevotellaceae, Succinivibrionaceae*, and *Lachnospiraceae* have been linked to low methane-emitting cows. Conversely, other OTUs assigned to the same genus were associated with high-emitting cows (Shabat et al., 2016; Danielsson et al., 2017; Tapio et al., 2017). Notably, a combination of phylogenetic and shotgun metagenomic analyses have been used to determine the functional characteristics of the rumen microbiome in low methane emitting cattle (Wallace et al., 2015; Shabat et al., 2016; Ramayo-Caldas et al., 2020). Moreover, most rumen bacteria are uncultured and physiologically unidentified (Creevey et al., 2014).

Therefore, this study aimed to investigate the association between rumen fermentation and rumen microbiota composition, focusing on methane production, and to elucidate the characteristics of bacteria found in low methane-producing cows to improve the understanding of rumen fermentation.

## 2 Materials and methods

### 2.1 Animals and sample collection

Thirteen lactating Holstein cows, which were maintained in the animal facility of the National Institute of Livestock and Grassland Science of NARO located in Tsukuba, in Ibaraki Prefecture, Japan, in late lactation stage (average 597 ± 56 kg) were selected for this study and fed total mixed ration (TMR) composed of corn silage and commercial concentrate pellet. The cows were housed individually in an air-conditioned animal facility (20°C, 60% humidity) for 3 weeks and fed TMR twice a day (at 09:45 and 19:00) to meet the energy requirement of the 2006 Japanese feeding standard for dairy cattle. The cows were milked twice daily before each feeding session. During the last 5 d, cows were placed in a whole-body respiration chamber to measure dry matter intake (DMI) and gaseous matter (methane, carbon dioxide, and oxygen) and to collect feces and urine for feed digestibility assay. Details of the respiration chamber system and representative sample processing procedures (feed, feces, and urine) have been previously described (Iwasaki et al., 1982; Shinkai et al., 2012). On the last day, rumen fluid was collected through the mouth before morning feeding using a rumen catheter (Sanshin Industrial, Yokohama, Japan), filtered through three layers of sterilized gauze, and pH was measured (edge, Hanna Instruments Inc., Smithfield, RI, USA). Microorganisms in the filtered rumen fluid (0.5 mL) were collected by centrifugation (14,000 × *g*, 5 min, 20°C) and stored at −20°C for DNA extraction. Other portions of the filtered rumen fluid were stored at −20 and −80°C for chemical analysis and bacterial isolation.

### 2.2 Chemical analysis

Rumen fluid stored at −20°C was centrifuged (14,000 × *g*, 5 min) and used for chemical analysis. SCFA concentrations were determined as described by Suzuki et al. (2021). Ammonia and lactate concentrations were determined spectrophotometrically using commercially available kits for ammonia (Wako Pure Chemical Industries, Osaka, Japan) and D- /L-lactic acid (Megazyme, Wicklow, Ireland), respectively.

### 2.3 DNA extraction, real-time PCR, and illumina sequencing

Total DNA was extracted from rumen fluid (0.5 mL) using the FastPrep FP100A bead-beating system with a Fast DNA SPIN Kit and Lysing Matrix E (MP Biomedicals, Solon, OH, USA) according to previously described procedures (Mitsumori et al., 2012), and purified using the ethanol precipitation method. DNA concentration was determined using Quant-iT dsDNA HS assay kit (Invitrogen). Thereafter, DNA samples were diluted with nuclease-free water to a concentration of 5 ng and 10 μg/μL for real-time PCR and Illumina sequencing, respectively. Real-time PCR was performed for total bacteria, ciliate protozoa, and methanogen genes (*mcrA*), and the relative abundances of protozoa and archaea to bacteria were calculated as described previously (Takizawa et al., 2023). For Illumina sequencing, 100 ng of DNA was amplified by PCR. The V3–V4 region of the bacterial 16S rRNA gene was amplified (20 PCR cycles) using KAPA HiFi HotStart ReadyMix (Roche, Basel, Switzerland) and primers (341F, 5′-CCTACGGGNGGCWGCAG-3′; 805R, 5′-GACTACHVGGGTATCTAATCC-3′) (Herlemann et al., 2011). PCR amplicon sequencing was performed on an Illumina MiSeq (Reagent kit v3, Illumina Inc. CA, USA) platform (Bioengineering Lab. Co., Ltd., Kanagawa, Japan). Details of the PCR primers and conditions for archaea, ciliates, and anaerobic fungi have been previously described (Kittelmann et al., 2013). Briefly, the primer sets Ar915aF and Ar1386R, RP841F and Reg1302R, and MN100F and MNGM2 without an adaptor were used for PCR amplification of 16S rRNA genes of archaea, ciliate 18S rRNA genes, and the anaerobic fungal ITS1 region, respectively. Specific amplification was confirmed via electrophoresis. The PCR products for archaea, protozoa, and fungi DNA were quantified as previously described, pooled at a ratio of 1:1:0.2 (Kittelmann et al., 2013), and subjected to Illumina sequencing (MiSeq 250bp × 2; New Zealand Genomics Ltd., Otago, NZ).

### 2.4 Phylogenetic analysis

Bacterial Illumina sequencing datasets (V3–V4 region of the 16S *rRNA* gene) were processed and analyzed using the QIIME2 version 2021.4 Quantitative Insights into Microbial Ecology software package (Bolyen et al., 2019). Briefly, primer sequences were trimmed using Cutadapt (Martin, 2011). Forward and reverse reads were merged using VSEARCH (Rognes et al., 2016), and the merged reads with a quality score < 20 were filtered. Using VSEARCH, the reads were dereplicated, clustered into OTUs with 97% similarity, and chimera-checked. The OTUs were assigned using the SILVA database version 138 99% OTUs (Quast et al., 2013), and the taxonomy of OTUs with specifically high abundance in the negative or positive PC1 score group was confirmed by searching against the NCBI rRNA/ITS databases using BLAST software. The mixture of sequence datasets for archaea, protozoa, and fungi was performed as previously described (Caporaso et al., 2010; Kittelmann et al., 2013) using QIIME2 (version 2023.9). As a significant proportion of the sequences obtained could not be merged, sequences over 200 bp in length were truncated such that the average quality score was over 20. Sequences of archaea, protozoa, and fungi were sorted using their respective forward primer sequences as barcodes and processed in the same way as those of bacteria. The databases used were for SILVA database version 138 99% OTUs for archaea and protozoa, and UNITE version 10.0 (Abarenkov et al., 2024) anaerobic fungi, respectively. Sequence data were deposited in DDBJ under accession numbers DRR360991–DRR361003 (DRA013965) for bacteria and DRR374802–DRR374814 (DRA014088) for archaea, protozoa, and fungi.

### 2.5 SU clone library construction and sequencing

Two 16S rRNA gene clone libraries were constructed to obtain 16S full-length sequences of OTU218 using 10 ng of DNAs isolated from cows 2011G and 2013Y. 16S rRNA genes were amplified on the TaKaRa Ex Taq HS PCR amplification system using the following primers: 27F (5′- AGAGTTTGATCMTGGCTCAG-3′) and 1492R (5′-GGYTACCTTGTTACGACTT-3′). The PCR conditions were as follows: 25 cycles of denaturation at 98°C (10 s), annealing at 55°C (15 s), and extension at 72°C (30 s). The PCR products were separated electrophoretically on an agarose gel (certified low-melt agarose, Bio-Rad) and purified using a QIAquick Gel Extraction Kit (QIAGEN, Hilden, Germany). Purified PCR amplicons were ligated into the vector pCR2.1 and inserted into One Shot TOP 10 (TA Cloning Kit, Invitrogen). A total of 94 and 91 plasmids for the libraries of 2011G and 2013Y, respectively, were isolated using the QIAprep Spin Miniprep kit (QIAGEN), quantified fluorometrically (Quant-iT dsDNA HS assay kit, Invitrogen), and sequenced by Eurofins Genomics Co., Ltd. (Sanger sequencing, Tokyo, Japan). The obtained sequences were compared with that of OTU218 and the isolated strains.

### 2.6 Isolation of *Prevotella* strains and identification

*Prevotella* strains, detected as OTU218, were isolated from the rumen of two cows maintained at the National Institute of Livestock and Grassland Science, according to previously described procedures (Shinkai et al., 2022). Briefly, rumen fluid was collected using a rumen catheter, sieved through three layers of surgical gauge, and diluted serially (10-fold) with an anaerobic dilution solution (Bryant and Burkey, 1953). Subsequently, diluted rumen bacteria were inoculated into modified YTR agar medium (Bryant and Burkey, 1953) to form roll tubes with a gas-tight Hungate anaerobic tube. The constituents of the modified YTR agar medium (per liter) include yeast extract, 1.2 g (Oxoid); Bacto peptone, 2 g (Difco); mineral solution I, 75 mL; mineral solution II, 75 mL; clarified rumen fluid, 300 mL; glucose, 1.5 g; cellobiose, 1.5 g; resazurin (0.1%), 10 mL; hemin solution (0.5 g /L), 10 mL; NaHCO3 (8%), 50 mL; L-cysteine hydrochloride, 3 g; bacto agar, 1.2 g (Difco); distilled water, 500 mL. The hemin solution was prepared as follows: 50 mg of hemin was dissolved in 1 ml of 1 N NaOH and made up to 100 ml with distilled water. Colonies that appeared after 48–72 h of incubation at 37°C were isolated. The 16S rRNA gene sequences of the strains were amplified using Takara Ex Taq HS (Takara) with the universal primers 27f and 1492r, purified using ExoSAP-IT (Applied Biosystems), and sequenced (Eurofins Genomics Co., Ltd.). Sequences of the novel *Prevotella* sp. obtained from Sanger sequencing, 16S *rRNA* gene clone library, and isolates were aligned with representative rumen bacteria using ClustalW (1.6) and subjected to phylogenetic analysis using the neighbor-joining method with 1,000 bootstrap replications (MEGA X) (Kumar et al., 2018).

### 2.7 Strains, growth condition, and fermentation product analysis

The type strains (*Prevotella ruminicola* B23^T^ (JCM 8259), *P. bryantii* B_1_4^T^ (DSM 11371), and *Butyrivibrio fibrisolvens* D1^T^ (JCM 6563) were obtained commercially. The type and isolated *Prevotella* strains were maintained in a modified YTR slant agar medium under anaerobic conditions with the Hungate tube. For growth monitoring and fermentation product analysis, the strains were grown on a glucose medium (modified PYG medium named DSMZ medium 104 by subtracting beef extract and Tween 80). Active strains were maintained by repetitive subculturing in glucose media as follows; 0.2 mL of bacterial cells at early to mid-log phase (OD_660_ = 0.3–0.5, OD_660_ = 0.1 in case of *P. ruminicola*) were transferred to fresh glucose media (5 mL). Bacterial growth was monitored every 10 min using an OD-Monitor C&T (TAITEC Co. Ltd.) installed in a shaking incubator (120 rpm, Bio-Shaker BR-40LF, TAITEC Co. Ltd.). The fermentation end products were determined after 44–48 h of incubation (stationary phase). The bacterial culture was repeated three times to obtain biological replicates. The organic acid profiles of the fermentation end-products were determined using a Shimadzu HPLC organic acid analysis system (Shimadzu Corp., Kyoto, Japan).

### 2.8 Statistical analysis

All variables, including methane production, DMI, rumen fermentation parameters, and milk production, were tested for normality and homogeneity using the Shapiro–Wilk normality test and Levene test, respectively. Normally distributed and homogeneous variables were compared using Student's *t*-test on R Commander (version 2.8-0). Welch's *t*-test (propionate % and SCFA ratio etc.) or Wilcoxon's rank sum test (ammonia concentration) were adopted based on the results of the normality and homogeneity check. Principal component analysis (PCA) was performed to elucide the interrelationships among rumen fermentation parameters using the prcomp () function in R package (version 3.6.1). The R packages ggbiplot (ver.0.55), and “vqv/ggbiplot” in “devtools” library were used for drawing correlation circle (*p* = 0.95), and adding normal probability ellipsoids. Rumen fermentation parameters were subjected to hierarchical cluster analysis (hclust) using the UPGMA method based on Z-score normalization, and the squared Euclidean distance was calculated to draw cluster dendrogram and heatmap using R. Spearman's rank correlation analysis was performed to determine the relationship among the data obtained in this study using R, with significance set at *p* < 0.05. Good's coverage index was employed to check the sequence coverage in Illumina sequences of bacteria, archaea, protozoa, and fungi. To assess the significance of community dissimilarity, non-metric multidimensional scaling (NMDS) based on Bray–Curtis dissimilarity was conducted using the R package vegan (ver. 2.6-2). Statistical significance of the clustering was analyzed using permutational multivariate analysis of variance (PERMANOVA) with the adonis2 function in the vegan package. The proportions of bacteria, archaea, protozoa, and fungi at the family and OTU levels were compared via PC1 score group and subjected to Wilcoxon's rank sum test using the coin package in R coin (version 1.4-2). OTUs that were specifically detected and undetected and that were significantly more and less abundant in the positive PC1 group were investigated. The relative composition of the fermentation products was compared among the strains. The relative proportion data of the pure culture fermentation products were subjected to normality test, Leven's test, and Welch's one-way ANOVA using R Commander, followed by Tukey's multiple comparison test for significant data. Data were considered statistically significant at *p* < 0.05.

## 3 Results

### 3.1 Animal production and rumen fermentation parameters

**Figure 1:**
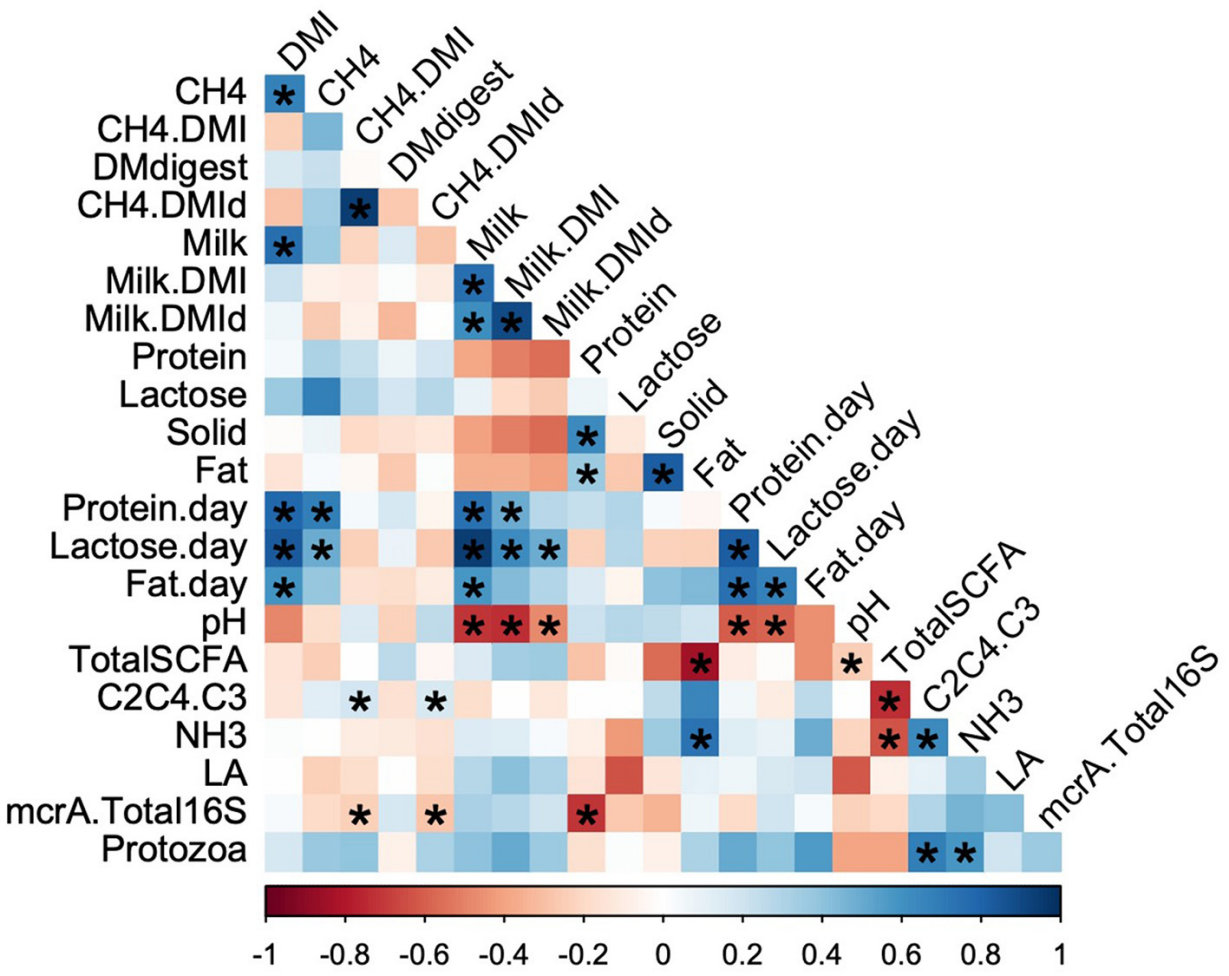
Correlation of rumen fermentation parameters with milk production. The color represents the Spearman's rank correlation coefficient values from −1, indicating a negative correlation (red), to 1, indicating a positive correlation (blue). Asterisks indicate statistical significance at *p* < 0.05. DMI, dry matter intake; DMdigest, dry matter digestibility; DMId, digested dry matter intake; SCFA, short chain fatty acid; LA, lactic acid; mcrA, mcrA gene; C2C4.C3, (C2+C4)/C3. The dotted line represents the slash.

The relationships among the data obtained in this study are shown in Figure 1, and all the collected datasets are listed in Supplementary Table S1. Spearman's rank correlation analysis indicated that DMI was positively correlated with daily methane and milk production, while total ruminal SCFA concentration was negatively correlated with SCFA ratio [(C2+C4)/C3]. However, DMI and methane production per unit of DMI (CH_4_/DMI) were poorly correlated, indicating that there were variations in both parameters among the cows.

**Figure 2:**
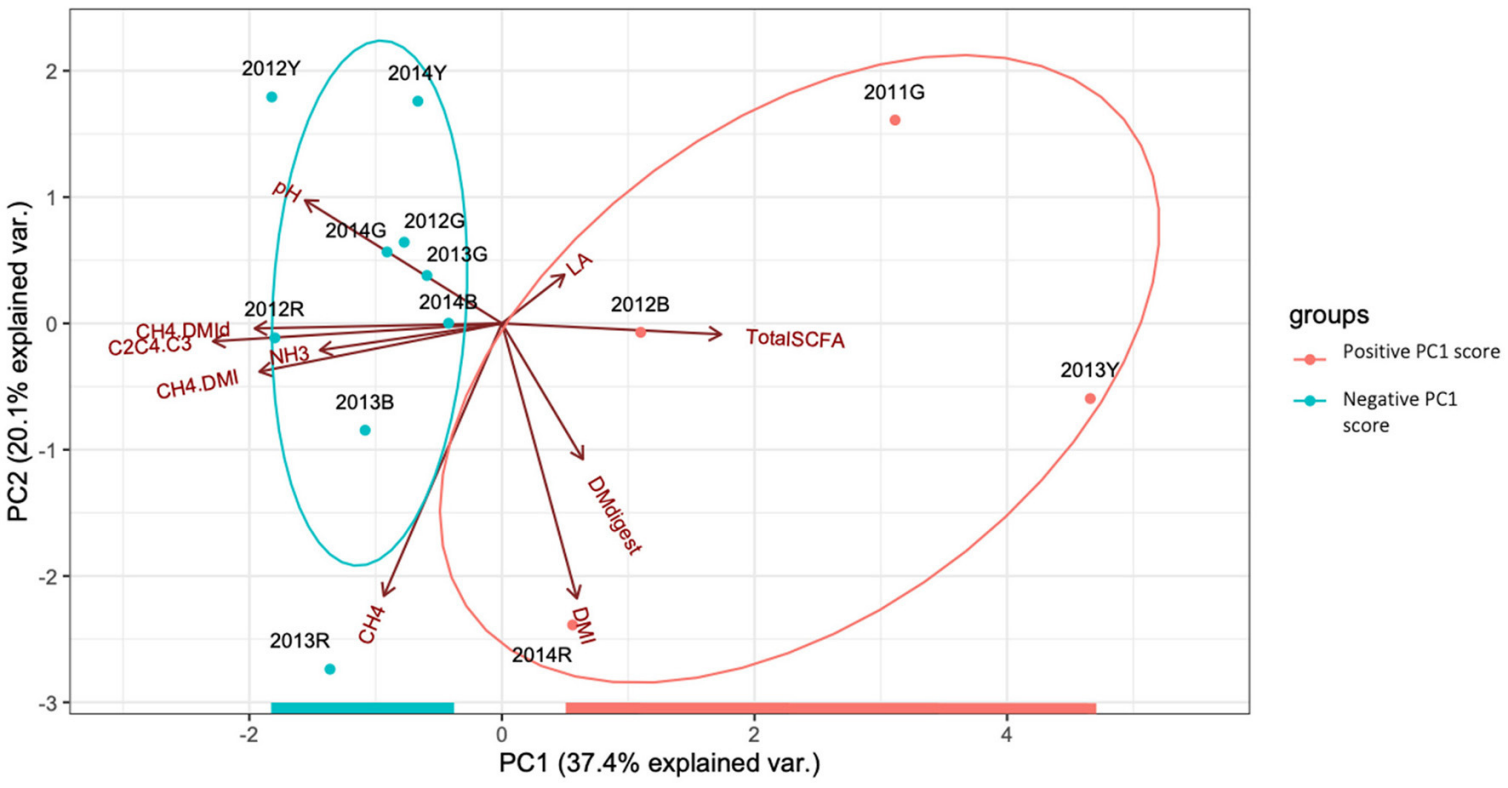
Principal component analysis (PCA) showing the interrelationship among rumen fermentation parameters. Normal probability ellipsoids for positive (orange) and negative (green) PC1 score groups are shown. DMI, dry matter intake; DMdigest, dry matter digestibility; DMId, digested dry matter intake; SCFA, short chain fatty acid; LA, lactic acid; C2C4.C3, (C2+C4)/C3. Dot represents slash.

PCA of rumen fermentation data, including DMI, CH_4_/DMI, and SCFA ratio, is shown in Figure 2. Approximately 57% of the total variance was explained by the PC1 and PC2. Based on the components of the PC1 and PC2 score data (Supplementary Table S2), the PC1 score was mainly defined by the SCFA ratio and methane production per unit of DMI, while that of PC2 was defined by the input and output amounts (DMI and daily methane production). To group cows based on rumen fermentation data, the PC1 score was used to focus on CH_4_/DMI and related parameters. Hierarchical clustering based on squared Euclidean distance provided support for grouping the cows based on rumen fermentation parameters (positive PC1 score group: 2011G, 2013Y, 2014R, and 2012 B; negative PC1 score group: the other nine cows) (Supplementary Figure S1).

Differences between the two PC1 score groups are summarized in Table 1. Specifically, CH_4_/DMI (25.0 vs. 28.2), CH_4_/digested DMI (36.9 vs. 43.2), and SCFA ratio (3.54 vs. 5.03) were lower in the positive PC1 score group than in the negative PC1 score group. Additionally, DM digestibility and propionate percentages were higher in the positive PC1 score group than in the negative PC1 score group. However, there were no significant differences in DMI, daily methane production, pH, and milk production between the two groups.

**Table 1:** Dry matter intake, rumen fermentation, milk production in the positive and negative PC1 score groups.

	**PC1 score group**			
**Item**	**Negative**	**Positive**	**SE**	***p-*** **value**
**Body weight and DMI**				
Body weight (kg)	591.2	612.6	20.8	0.543
DMI (kg)	18.9	20.4	1.17	0.267
DM digestibility (%)	65.3	67.7	1.08	0.033
**Rumen fermentation**				
Methane (L)	533.4	506.1	35.6	0.518
Methane/DMI (L/kg)	28.2	25.0	1.65	0.021
Methane/DMI digested (L/kg)	43.2	36.9	4.70	0.003
pH	7.10	6.85	0.22	0.149
Total SCFA (mM)	65.0	78.9	8.37	0.195
Acetate (%)	70.9	66.6	2.93	0.059
Propionate (%)	16.3	23.0	2.56	0.077
Butyrate (%)	10.8	8.8	0.74	0.022
SCFA ratio [(C2+C4)/C3]	5.03	3.54	1.15	0.079
Lactate (mM)	0.74	0.80	0.10	0.709
Ammonium-nitrogen (mg/dL)	5.73	3.08	1.68	0.213
**Milk production**				
Milk (kg)	23.4	25.6	2.43	0.537
Milk / DMI (kg/kg)	1.24	1.25	0.07	0.856
Protein (%)	3.51	3.48	0.14	0.834
Lactose (%)	4.64	4.68	0.06	0.653
Fat (%)	4.19	4.05	0.35	0.671

DMI, dry matter intake; SCFA, short chain fatty acid.

**Figure 3:**
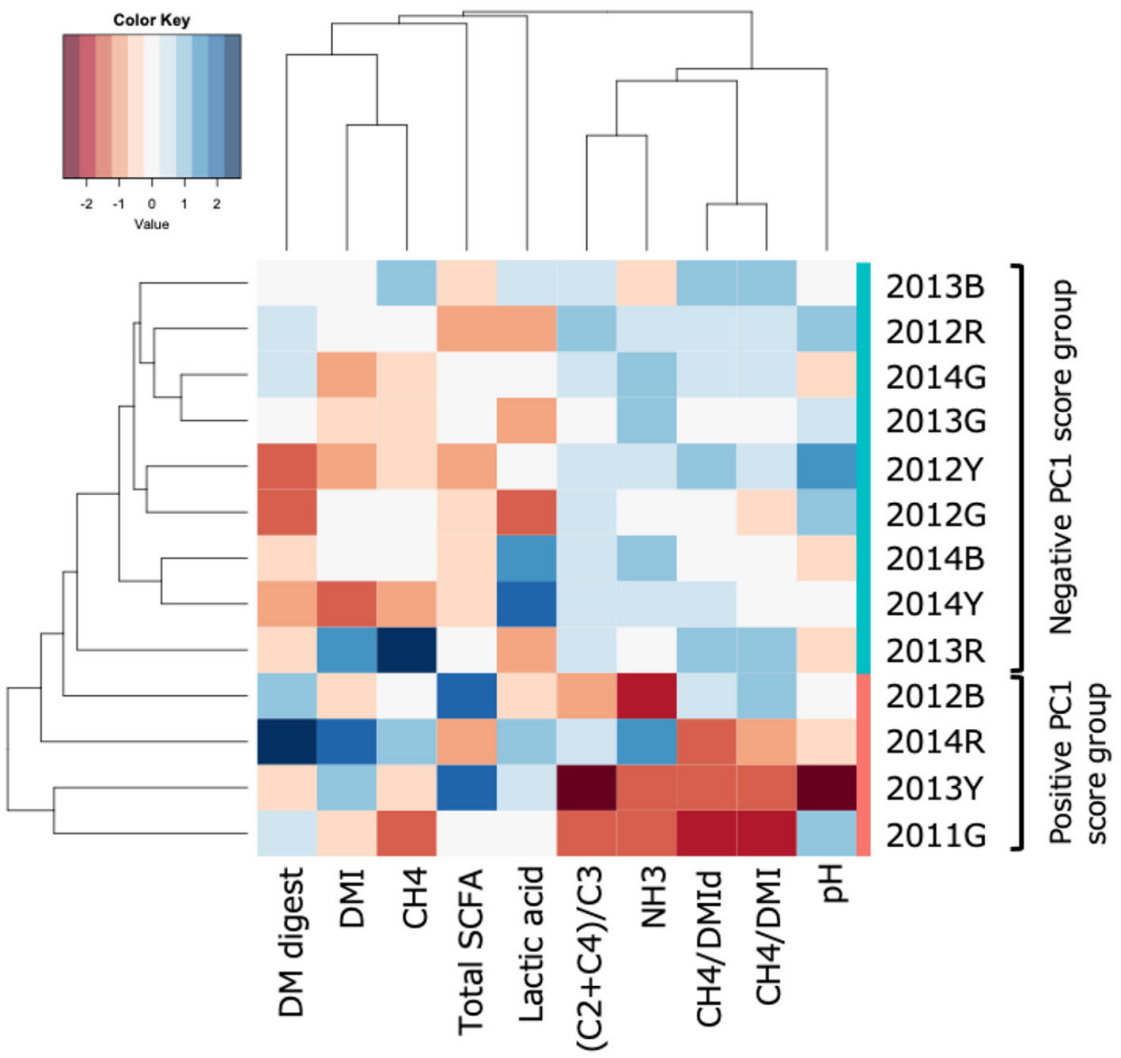
Heatmap of the rumen fermentation parameters by individual cows. A cluster dendrogram was constructed via hierarchical cluster analysis (hclust) using the UPGMA method based on Z-score-normalized data.

Heatmap of the rumen fermentation parameters of individual cows is shown in Figure 3. Cluster analysis based on normalized rumen fermentation datasets showed a relationship among the fermentation parameters, with a close relationship observed between DMI and daily methane production. Similarly, methane production per unit of DMI or apparently digested DMI was associated with SCFA ratio [(C2+C4)/C3].

### 3.2 Microbial community analysis

**Figure 4:**
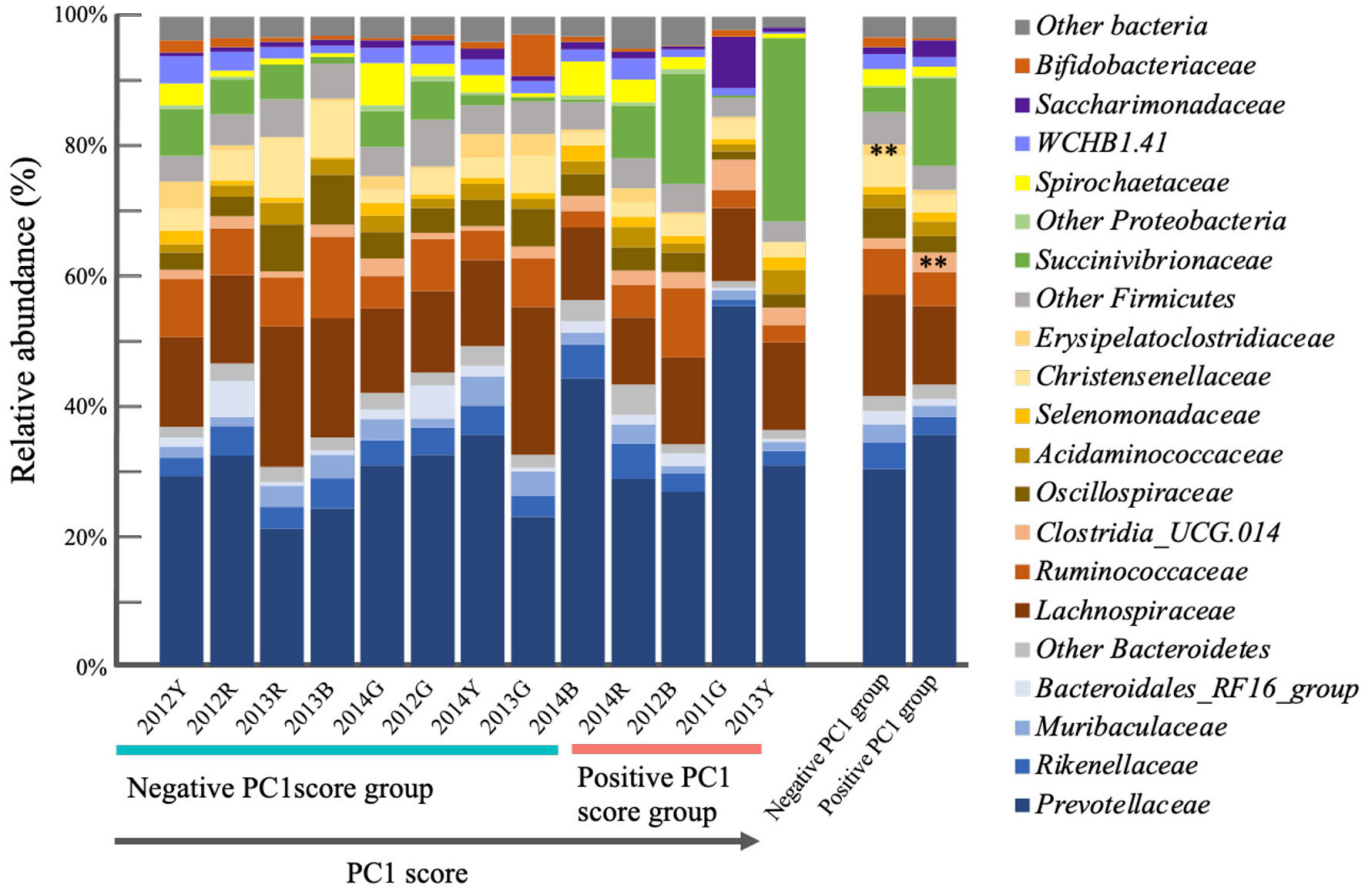
Relative composition of bacteria in the positive and negative PC1 score cows. **p < 0.05.

Good's coverage index, a parameter of the sequencing depth was 98.937–99.600, 99.318–99.984, 99.588–99.985, and 99.487–99.977 for bacterial, archaeal, protozoan, and fungal sequences, respectively. Bacterial communities of the positive and negative PC1 score groups were significantly different (*P* = 0.03996). The differences in bacterial composition are shown in Figure 4. At the phyla level, the most abundant rumen bacterial phyla in the positive and negative PC1 score groups were Bacteroidetes (43.2% vs. 41.6%), Firmicutes (33.8% vs. 43.7%), and Proteobacteria (13.8% vs. 4.2 %). At family the level, Prevotellaceae (35.5% vs. 30.4%), Lachnospiraceae (12.2% vs. 15.8%), Succinivibrionaceae (13.4% vs. 3.7%), Ruminococcaceae (5.2% vs. 7.1%), Clostridia_UCG.014 (3.0% vs. 1.6%, *P* < 0.05), Rikenellaceae (2.8% vs. 4.1%), Christensenellaceae (2.7% vs. 4.7%), Oscillospiraceae (2.5% vs. 4.6%), Saccharimonadaceae (2.5% vs. 1.0%), Acidaminococcaceae (2.3% vs. 2.1%), Muribaculaceae (1.7% vs. 2.7%), family WCHB1.41 (1.5% vs. 2.4%), Spirochaetaceae (1.5% vs. 2.5%), Bacteroidales_RF16_group (1.1% vs. 2.1%), and Erysipelotrichaceae (0.003% vs. 0.015%, *p* < 0.05) were detected in the positive and negative PC1 score groups. Among 883 bacterial OTUs detected, 40 OTUs, including phyla Bacteroidetes (15 OTUs including *Prevotella ruminicola* and *P. bryantii*), Firmicutes (23 OTUs, including *Ruminococcus flavefaciens* and *Ruminococcus bacterium_YRD2003*), and *Patescibacteria* (2 OTUs) were common to both groups. The representative bacterial OTUs in the PC1 score group are shown in Table 2. *Prevotella* (OTU218, *p* < 0.1) and *Succinivibrionaceae_UCG-001* (OTU851, *p* < 0.05) and other 12 bacterial OTUs were higher in the positive PC1 score group than in the negative PC1 score group. Specifically, OTU218 and 851 were abundantly detected in cows with positive PC1 scores (Table 2). Full-length 16S *rRNA* gene sequences were assigned to uncultured *Prevotella* sp. and uncultured *Succinivibrionaceae* (56 and 14 clones for OTU218 and OTU851, respectively).

**Table 2:** Relative proportion of detected OTUs in PC1 score group.

	**Taxonomy**			**PC1 score group (%)**		
**OUT No**.	**Phylum**	**Family**	**Genus**	**Negative**	**Positive**	***p*** **-value**
**Negative PC1 score group-representative OTU**						
OTU198	Firmicutes	Oscillospiraceae	*NK4A214_group*	2.86	1.31	0.006
OTU856	Firmicutes	Ruminococcaceae	*Ruminococcus*	2.53	0.32	0.034
OTU469	Bacteroidetes	Prevotellaceae	*Prevotella*	0.70	0.12	0.020
OTU31	Firmicutes	Christensenellaceae	*Christensenellaceae_R-7_group*	0.39	0.18	0.034
OTU611	Verrucomicrobiota	WCHB1-41	*WCHB1-41*	0.35	0.10	0.020
OTU349	Firmicutes	Ruminococcaceae	*Ruminococcus*	0.34	0.04	0.032
OTU430	Bacteroidetes	Prevotellaceae	*Prevotella*	0.30	0.02	0.045
OTU285	Bacteroidetes	Muribaculaceae	*Muribaculaceae*	0.27	0.05	0.032
OTU475	Firmicutes	Lachnospiraceae	*Moryella*	0.23	0.07	0.006
OTU823	Firmicutes	Lachnospiraceae	*Oribacterium*	0.18	0.03	0.018
OTU740	Firmicutes	Lachnospiraceae	*Lachnospiraceae_ND3007_group*	0.18	0.03	0.035
OTU700	Firmicutes	Christensenellaceae	*Christensenellaceae_R-7_group*	0.15	0.02	0.010
**Positive PC1 score group-representative OTU**						
OTU218	Bacteroidetes	Prevotellaceae	*Prevotella*	0.01	15.48	0.077
OTU851	Proteobacteria	Succinivibrionaceae	*Succinivibrionaceae_UCG-001*	< 0.01	7.74	0.014
OTU512	Bacteroidetes	Prevotellaceae	*Prevotella*	0.01	0.58	0.077
OTU597	Firmicutes	Selenomonadaceae	*Schwartzia*	0.13	0.47	0.010
OTU646	Bacteroidetes	F082	*F082*	0.01	0.27	0.001
OTU92	Patescibacteria	Saccharimonadaceae	*Candidatus_Saccharimonas*	0.01	0.23	0.014
OTU227	Bacteroidetes	Prevotellaceae	*Uncultured*	0.01	0.16	0.077
OTU186	Bacteroidetes	Prevotellaceae	*Prevotella*	< 0.01	0.15	0.001
OTU170	Bacteroidetes	Bacteroidales_RF16_group	*Bacteroidales_RF16_group*	0.01	0.14	0.077
OTU240	Firmicutes	[Eubacterium] _coprostanoligenes_group	*[Eubacterium]_coprostanoligenes _group*	0.02	0.10	0.029
OTU753	Bacteroidetes	Rikenellaceae	*U29-B03*	0.02	0.10	0.039
OTU96	Firmicutes	Lachnospiraceae	*-*	0.01	0.08	0.039
OTU25	Firmicutes	Clostridia_UCG-014	*Clostridia_UCG-014*	< 0.01	0.07	0.014
OTU433	Bacteroidetes	Prevotellaceae	*Prevotella*	0.01	0.06	0.018

Bacterial OTUs accounting more than 0.05% are shown. OTU, operational taxonomic unit.

No significant differences in archaeal, protozoal, and fungal communities between the positive and negative PC1 score groups were obtained with the dissimilarity analysis (*P* = 0.2188, 0.1948, and 0.7842, respectively). Their community compositions are shown in Supplementary Figures S2a–S2c. The most abundant archaeal groups in the positive and negative PC1 score groups were *Methanobrevibacter*_D_1148 (49.3% vs. 39.2%), followed by *Methanobrevibacter*_A (36.5% vs. 36.3%), and *Unclassified Methanobacteriaceae* (7.1% vs. 20.5%). *Entodinium* and *Piromyces* were the predominant protozoans and fungi, respectively, in both groups. However, there were significant differences in archaeal, protozoal, and fungal compositions between the two PC1 score groups.

### 3.3 Isolation and the fermentation products

**Figure 5:**
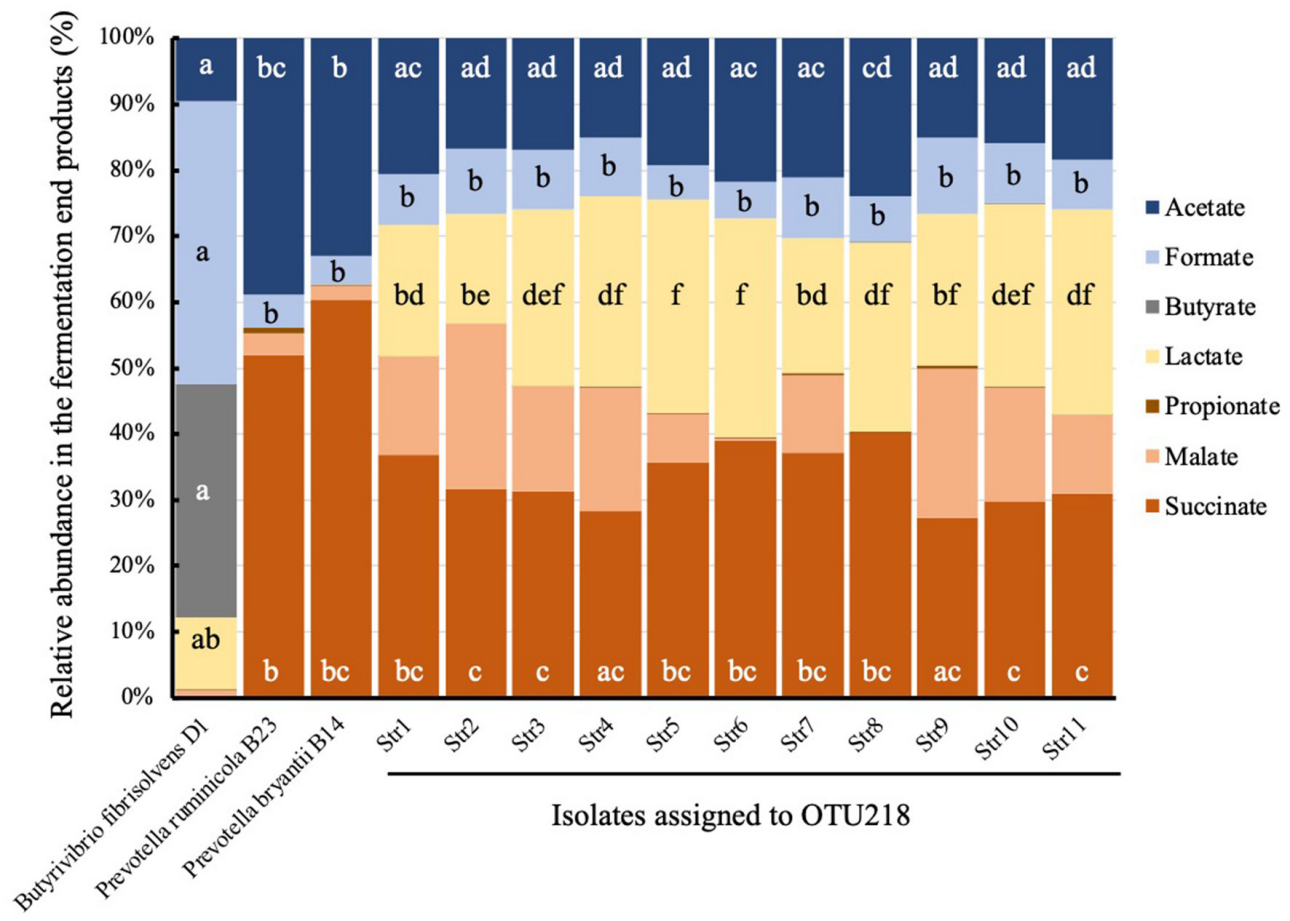
The molar proportion of fermentation end products in the culture of the isolate assigned to OTU218. Succinate (orange), malate (light orange), lactate (yellow), acetate (blue), and formate (light blue) are produced from glucose. Means with different superscripts across the same fermentation products differ significantly at *p* < 0.05.

Eleven strains assigned to the sequences of OTU218 and full length SSU clones were isolated from 262 colonies (Supplementary Figure S3). All 11 isolates were classified into the genus *Prevotella* based on the 16S *rRNA* gene sequences, with < 89.6% sequence similarity compared with the known *Prevotella* species. Succinate, lactate, acetate, formate, and malate were detected as fermentation end-products when the strains were grown on glucose (Figure 5). A comparison of the fermentation end products of the 11 isolates and *P. ruminicola* showed that the novel isolates produced more lactate (26.2% vs. 0%), malate (13.2% vs. 3.4%), and formate (8.2% vs. 5.0%), and less succinate (33.5% vs. 51.1%) and acetate (18.6% vs. 38.3%) than *P. ruminicola*.

## 4 Discussion

Ruminal methane production is a complex process affected by DMI, feed composition, rumen microbiota and their fermentation, lactation stage, host genetics, and the environment (Tapio et al., 2017; Difford et al., 2018; Lyons et al., 2018; Greening et al., 2019). Host genetics research uses spot methane measurement data to obtain animal data (Wallace et al., 2019). Although only a limited number of animals can be tested, respiration chambers can be used to collect accurate methane volumes under fixed feeding management (time and amount), sampling time, and environmental conditions (Lyons et al., 2018). Some studies have used PCA or canonical correspondence analysis and its score rank to classify ruminants according to the microbial type (Kittelmann et al., 2014; Belanche et al., 2015; Danielsson et al., 2017). In the present study, rumen fermentation efficiency and products were evaluated based on PCA of rumen fermentation parameters (Denman et al., 2015). Specifically, the relative values of the SCFA ratio [(C2 + C4)/C3] and methane production per unit of DMI and/or apparently digested DMI were defined by the PCA score (Supplementary Table S2). Hydrogen or metabolic hydrogen generated during rumen fermentation can be consumed by microbes and microbial products, including methane, propionate, and biomass (Ungerfeld, 2020). Methane and propionate syntheses can function as the main hydrogen sinks in the rumen, and a strong negative correlation can be detected between these two processes (Janssen, 2010; Brask et al., 2015; Williams et al., 2019). In the present study, the PC1 score was mainly affected by methane production per unit of apparently digested DMI and SCFA ratios, likely representing a complex hydrogen balance during ruminal fermentation. DMI and methane production contributed more to the PC2 score (Figure 2, Supplementary Table S2), and both factors were positively correlated (Figure 1) (Shibata and Terada, 2010). Additionally, the positive PC1 score group had higher DM digestibility than the negative PC1 score group (Table 1). Previous studies have indicated that feed passage rate, which affects the amount of digested DMI, is an important determinant of variation in methane production among animals (Janssen, 2010; Brask et al., 2015; Kamke et al., 2016).

Methane per unit of digested DMI and DMI were positively correlated with SCFA ratio and negatively correlated with *mcrA* expression per unit of bacteria (Figure 1). Methanogenic Archaea are closely associated with enteric methane production. The observed association between *mcrA* expression or the Archaea:Bacteria ratio and methane emission in the present study was consistent with previous findings (Roehe et al., 2016; Auffret et al., 2018). In contrast, weak or no correlation has been observed between *mcrA* expression or the Archaea:Bacteria ratio and methane emission (Kittelmann et al., 2014). Methanogenesis, methanogenic populations, and acetate:propionate ratios are highly correlated at low pH owing to the high sensitivity of methanogens to acidic conditions (Lana et al., 1998). Similarly, protozoan populations are sensitive to low pH. Considering the pH values and microbial abundance observed in this study, cows may maintain normal pH conditions under strict feeding management. Fauna-free sheep have been reported to produce lower amount of methane (Belanche et al., 2015). A meta-analysis revealed that 21% of experiments reported variations in methane emissions without changes in protozoa numbers, whereas methane mitigation with protozoa reduction was reported in lipid-fed animals (Guyader et al., 2014). Therefore, it could be speculated that the protozoan population may not have directly affected methane production in the present study, but indirectly regulated it by changing the SCFA ratio (Figure 1).

Additionally, differences were observed in rumen bacterial composition between the positive PC1 and negative PC1 groups (Figure 4). At the family revel, Erysipelotrichaceae had a higher abundance in the positive PC1 score group (0.003% vs. 0.015%, *p* < 0.05) than in the negative PC1 score group. The family Erysipelotrichaceae includes lactate producers, represented by *Sharpea*, which is abundant in low-methane-emitting sheep (Kamke et al., 2016). However, Erysipelotrichaceae abundance seemed to have little impact on rumen fermentation in the positive PC1 score group. OTU clustering analysis identified PC1 group-specific bacterial OTUs (Table 2), among which *Prevotella* OTUs were detected in both the positive and negative PC1 score groups (OTUs 469, 430, 218, 512, 227, 186, and 433), which was consistent with previous findings (Danielsson et al., 2017; Liu et al., 2017). Various OTUs of the genus *Prevotella* have shown negative or positive correlations with methane production, rumen fermentation, feed efficiency, milk, and milk fat yields (Tapio et al., 2017; Schären et al., 2018). Ruminal *Prevotella* is associate with propionate production and play an important role in ruminal metabolism by utilizing non-cellulosic plant polysaccharides and starches (Denman et al., 2015; Accetto and Avguštin, 2019; Betancur-Murillo et al., 2022). Moreover, species of the genus *Prevotella* (>60 species) show considerable variation in substrate preference and function (Mizrahi and Jami, 2018; Accetto and Avguštin, 2019). Although several phylogenetically different *Prevotella* OTUs have been detected in the rumen of cows with high and low methane emissions, it is difficult to understand the functional differences between these OTUs because most are uncultured (Danielsson et al., 2017; Liu et al., 2017; Mizrahi and Jami, 2018).

The isolates assigned to OTU218 were abundantly detected in the positive PC1 score group (0.01% vs. 15.48%, *p* < 0.1). Moreover, OTU218 is a phylogenetically novel species based on the 16S rRNA sequencing. Therefore, we compared OTU218 to other *Prevotella* species based on phylogenetic, physiological, and genomic analyses, and named it *Prevotella lacticifex* (Shinkai et al., 2022). The details of the genomic information of *P. lacticifex* can be found in GenBank/EMBL/DDBJ (accession No. LC639953 through LC639963 for the 16S rRNA gene and BioProject accession No. PRJDB12066 for the whole genome). Unlike other ruminal *Prevotella* species (*P. ruminicola* and *P. bryantii*), *P. lacticifex* produces significant amounts of lactic acid from glucose (Figure 5). The profiles of the fermentation end-products of *P. ruminicola, P. bryantii*, and *Butyrivibrio fibrisolvens* were similar to those reported in previous studies, except for one study that reported propionate production by *P. ruminicola* (Emerson and Weimer, 2017; Seshadri et al., 2018). Lactic acid production and utilization are characteristic traits of low-methane emitters, and two types of microbiota with low methane yields have been reported (Kittelmann et al., 2014). Kamke et al. (2016) found that L-(+)-lactate dehydrogenase genes associated with lactate formation from pyruvate were highly abundant in low-methane emitting sheep, and it was concluded that the increased lactate production and the corresponding increase in lactate conversion to butyrate resulted in a decrease in methane production. Similar microbiome differences were observed in low-methane-emitting dairy cows (Shabat et al., 2016). Propionate precursors have been evaluated as alternative electron acceptors for methanogenesis during rumen fermentation (Newbold et al., 2005). *P. lacticifex* isolates produce propionate precursors, including succinate, malate, and lactate. Lactate may be generated by lactate utilizers detected the in positive PC1 score group, such as the families of Selenomonadaceae (*p* < 0.05) and Saccharimonadaceae, a possible lactate utilizer formerly known as TM7 (Bor et al., 2019). In a previous study, it was found that *Prevotella*, but not *P. lacticifex* and *Selenomonas* sp. contributed to lactate conversion to succinate via the randomizing succinate pathway (Denman et al., 2015). A study on hydrogen production and consumption indicated that the consumption of hydrogen by [NiFe]-hydrogenases of methanogens and selenomonads in sheep suggests the importance of selenomonads in direct hydrogen consumption (Greening et al., 2019). Based on these findings, the abundance of the propionate precursor-producing *P. lacticifex* and its utilizers in the positive PC1 score group contributed to the higher propionate production and lower methane production observed in the present study.

Furthermore, a relationship between enteric methane production and uncharacterized Succcinivibrionaceae as well as *Prevotella* spp. has been suggested in both dairy and beef cattle (Wallace et al., 2015; Danielsson et al., 2017; Tapio et al., 2017). The family Succinivibrionaceae includes starch fermenters such as *S. dextrinosolvens, Ruminobacter amylophylus*, and a strain isolated from the tammar wallaby that produces succinate and acetate (Pope et al., 2011). Moreover, an uncharacterized Succinivibrionaceae, which is related to low methane production, is yet to be cultured and identified. Liu et al. (2017) reported a strong correlation between *Prevotella* and *Methanobrevibacter* in young Heifers (9–10 months), which was replaced by a correlation between *Succinivibrio* and *Methanobrevibacter* in older cows (96–120 months). A co-culture study demonstrated that the specific interaction between *S. dextrinosolvens* H5 and methanogens enhanced the growth of *Methanomassillicoccales* but inhibited *Methaobrevibacter gottschalkii* growth (Kamke et al., 2017). A negative correlation exists between uncharacterized Succcinivibrionaceae and methanogens (McCabe et al., 2015); therefore, further studies are necessary to elucidate these interdomain interactions to improve the understanding of enteric methane production.

## 5 Conclusions

This study attempted to identify novel rumen microbes associated with low methane emission in Holstein cows. Cows with low methane and high propionate production showed higher DM digestibility and total SCFA concentration without affecting DMI and milk production. Additionally, there were significant differences in the abundance of OTUs assigned to uncultured *Prevotella* sp., *Succinivibrio*, and other 12 bacterial phylotypes between low methane and high methane emitting cows. Moreover, a previously uncultured novel *Prevotella* sp. with a lactate-producing phenotype was detected, with a higher abundance in low methane emitting cows. Overall, this study provides evidence that *Prevotella* may be related to low methane and high propionate-producing cows. However, further research is required to understand the microbial relationships and metabolic processes involved in the mitigation of enteric methane.

## Data availability statement

All data generated or analyzed during this study are included in this published article and its Supplementary information files. Sequence data are deposited in DDBJ under accession numbers DRR360991 through DRR361003 (DRA013963 through DRA013975) for bacteria and DRR3748002 through DRR374814 (DRA014088) for archaea, protozoa, and fungi. The type strain of *Prevotella lacticifex* was obtained from JCM and DSM (JCM 34664 and DSM 112675, respectively).

## Ethics statement

The animal study was approved by the National Agricultural and Food Research Organization. The study was conducted in accordance with the local legislation and institutional requirements.

## Author contributions

TS: Conceptualization, Data curation, Formal analysis, Funding acquisition, Investigation, Methodology, Project administration, Validation, Visualization, Writing – original draft, Writing – review & editing. ST: Data curation, Formal analysis, Investigation, Methodology, Validation, Visualization, Writing – review & editing. OE: Conceptualization, Data curation, Formal analysis, Funding acquisition, Investigation, Methodology, Project administration, Resources, Validation, Writing – review & editing. KH: Data curation, Formal analysis, Investigation, Methodology, Validation, Writing – review & editing. HO: Data curation, Formal analysis, Methodology, Resources, Validation, Writing – review & editing. MM: Conceptualization, Data curation, Funding acquisition, Investigation, Methodology, Project administration, Writing – review & editing.
