# Supplementary material for: Characteristics of rumen microbiota and Prevotella isolates found in high propionate and low methane-producing dairy cows
Source: Front Microbiol. 2024 Jun 3;15:1404991. doi: 10.3389/fmicb.2024.1404991 (PMC11180796; doi:10.3389/fmicb.2024.1404991)
Supplement: Supplementary file 1 [file Data_Sheet_1.docx]

Supplementary Material

Characteristics of rumen microbiota and *Prevotella* isolates found in high propionate and low methane-producing dairy cows

Takumi Shinkai*, Shuhei Takizawa, Osamu Enishi, Koji Higuchi, Hideyuki Ohmori, and Makoto Mitsumori

NARO Institute of Livestock and Grassland Science, Ibaraki 305-0901, Japan

*** Correspondence:**Takumi Shinkai

Phone number: +81-29-838-8660

[tshinkai@affrc.go.jp](mailto:tshinkai@affrc.go.jp)

**Table S2.** Components of PC scores of rumen fermentation parameters in principal component analysis (PCA) are shown in Figure 2.

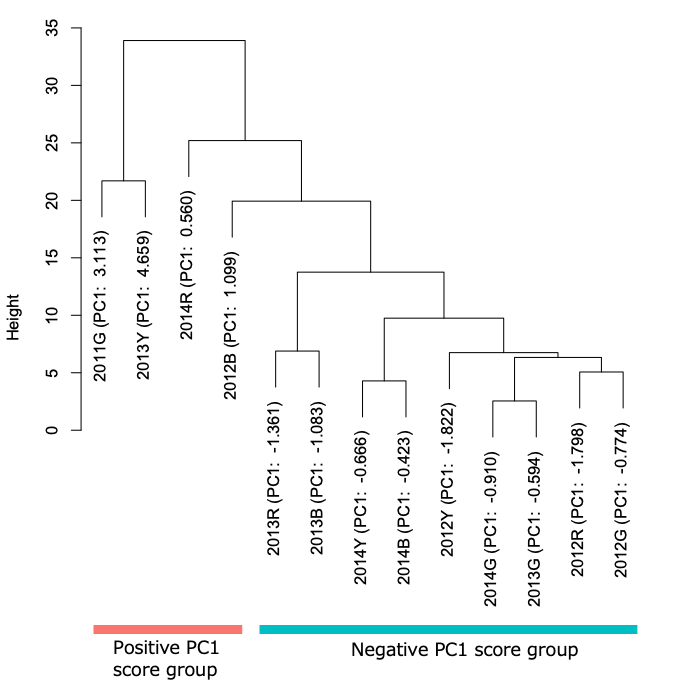


**Figure S1.** Cluster dendrogram with squared Euclidean distance. Rumen fermentation data were normalized using the Z-score method for scaling. The squared Euclidean distance was calculated and the average (UPGMA) method was used for hierarchical cluster analysis (hclust).

**(a)**


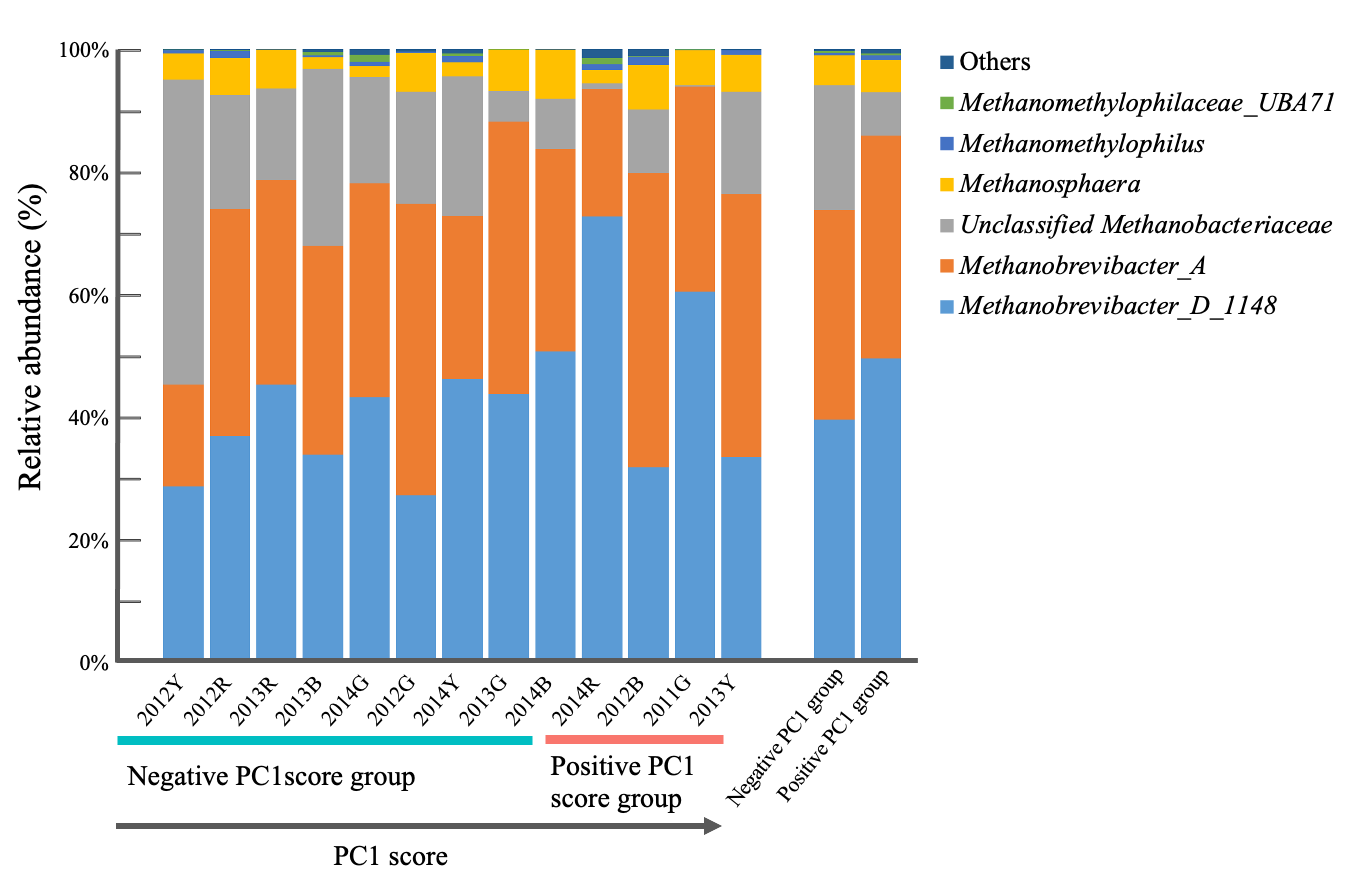


**(b)**


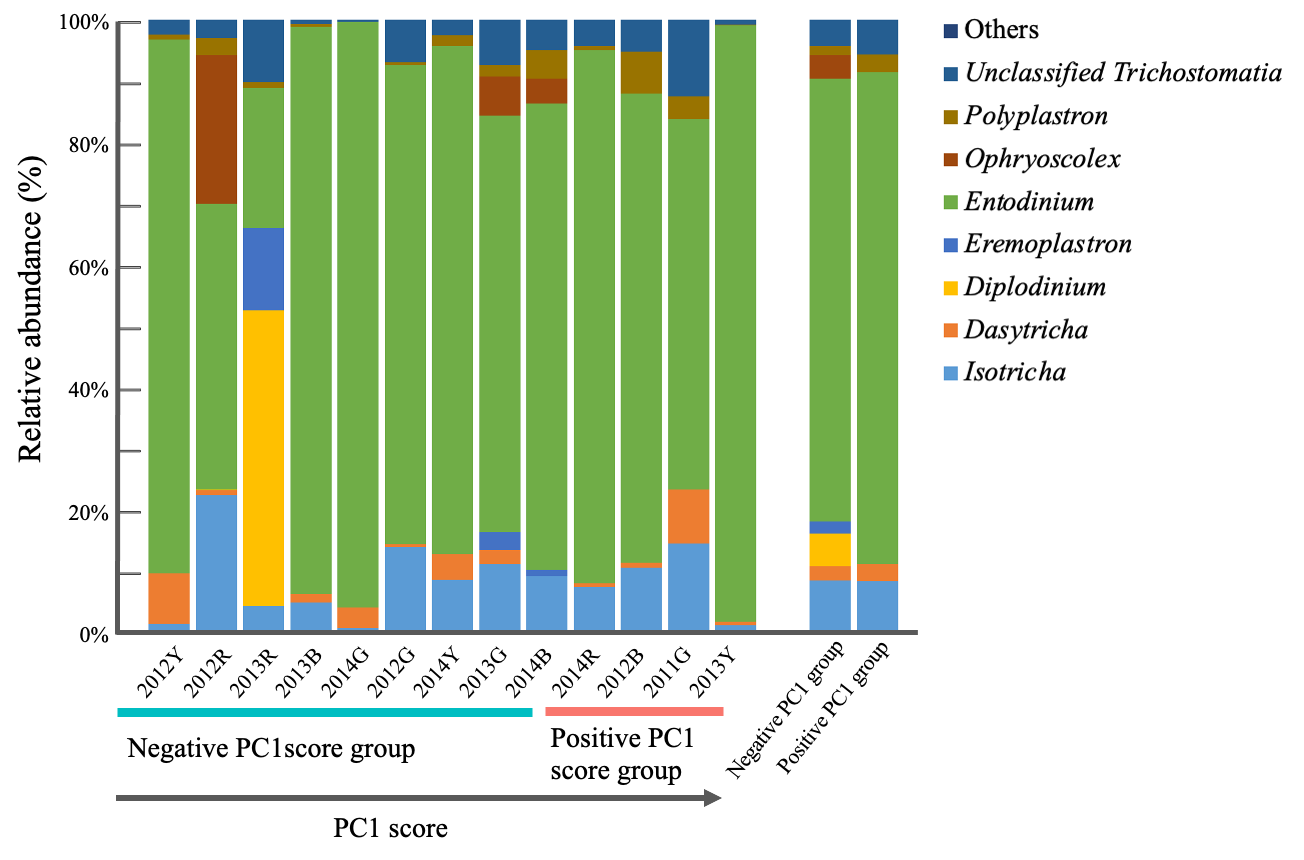


**(c)**


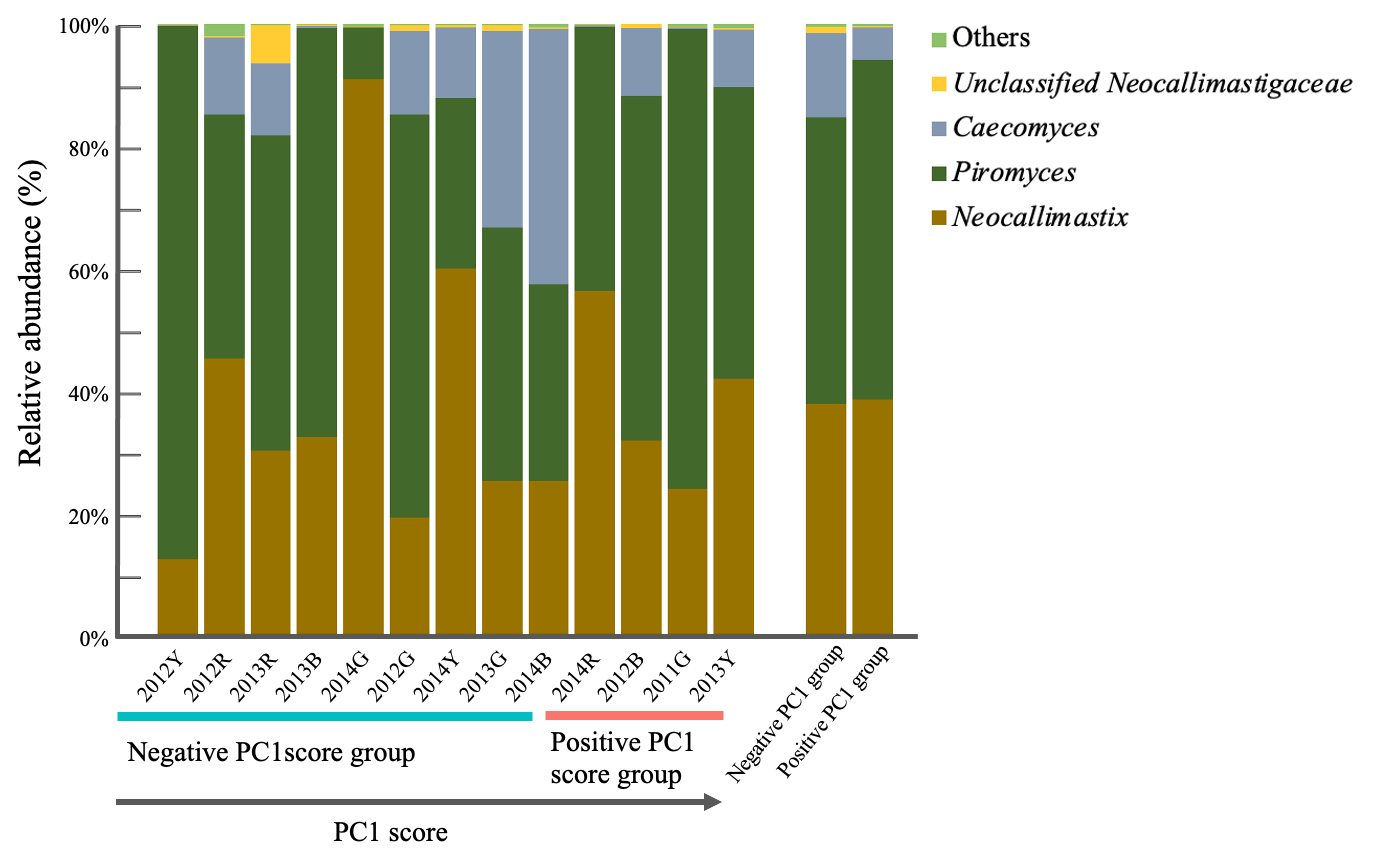


**Figure S2.** Relative compositions of archaea (a), ciliate protozoa (b), and fungi (c) in cows with positive and negative PC1 scores. *; *p* < 0.10


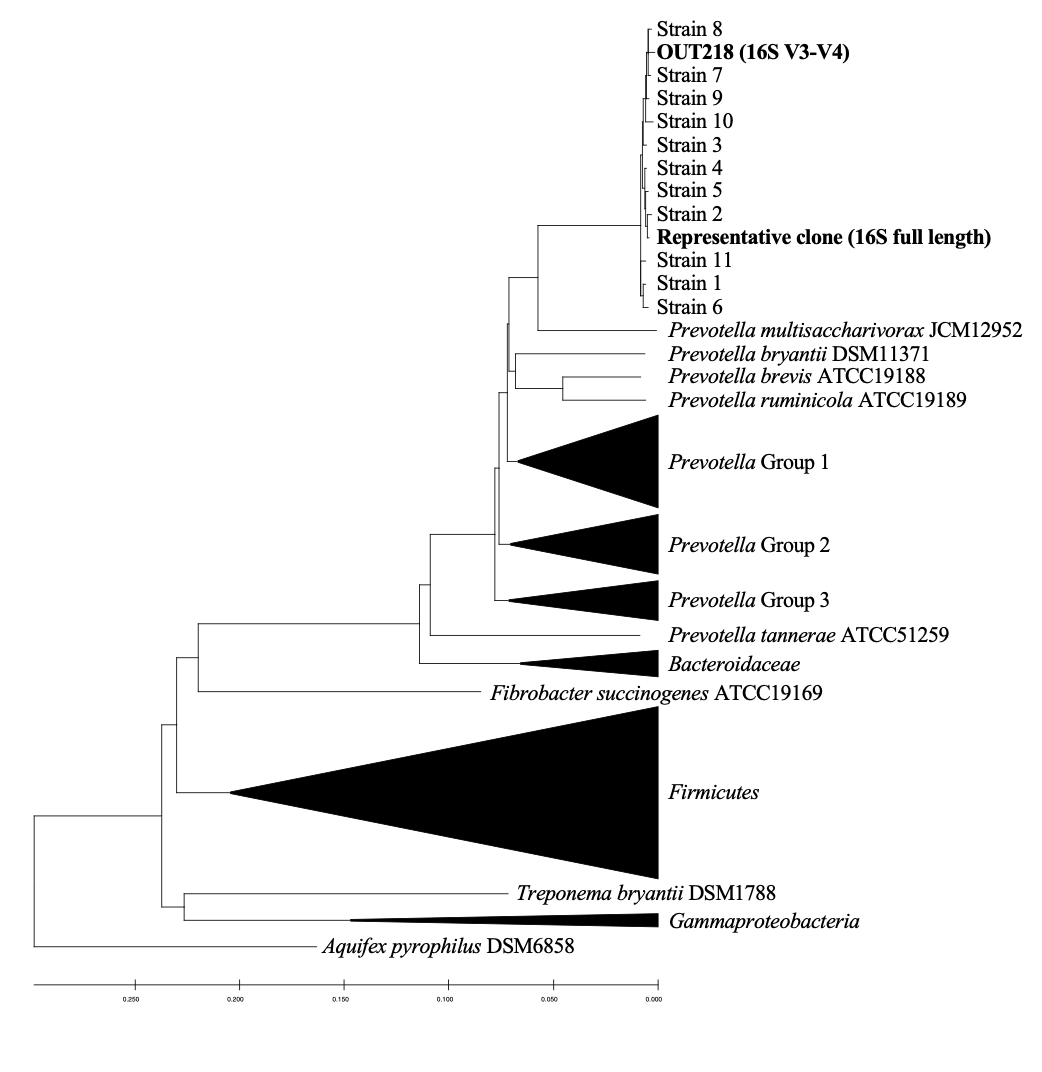


**Figure S3. Phylogenetic relationship of Prevotella species, representative 16S rRNA sequences and isolates based on 16S rRNA gene sequences.** A phylogenetic tree generated using the neighbor-joining method represented the phylogenetic relationship between detected OTU218 and the full length 16S rRNA gene clone and isolated 11 strains. The sequence of *Aquifex pyrophilus* (accession number: NR029172) was used as an outgroup. The numbers (≥80) given at the nodes show bootstrap values representing the confidence percentage given from 1,000 trees. The 16S rRNA gene sequences of the isolated strains were deposited in DDBJ under the accession numbers LC639953–LC639963.
